# Phosphate Concentrations and Modifying Factors in Healthy Children From 12 to 24 Months of Age

**DOI:** 10.1210/clinem/dgab495

**Published:** 2021-07-02

**Authors:** Laura Koljonen, Maria Enlund-Cerullo, Helena Hauta-alus, Elisa Holmlund-Suila, Saara Valkama, Jenni Rosendahl, Sture Andersson, Minna Pekkinen, Outi Mäkitie

**Affiliations:** 1 Folkhälsan Research Center, 00290 Helsinki, Finland; 2 Research Program for Clinical and Molecular Metabolism, Faculty of Medicine, University of Helsinki, 00014 Helsinki, Finland; 3 Children’s Hospital, Pediatric Research Center, University of Helsinki and Helsinki University Hospital, 00014 Helsinki, Finland; 4 National Institute for Health and Welfare (THL), 00271 Helsinki, Finland; 5 PEDEGO Research Unit, MRC Oulu, Oulu University Hospital and University of Oulu, 90014 Oulu,Finland; 6 Department of Molecular Medicine and Surgery, Karolinska Institutet, and Clinical Genetics, Karolinska University Hospital, 17176 Stockholm, Sweden

**Keywords:** clinical trial, vitamin D, mineral homeostasis, hypophosphatemia, phosphate

## Abstract

**Context:**

Phosphate homeostasis and its modifiers in early childhood are inadequately characterized.

**Objective:**

To determine physiological plasma phosphate concentration and modifying factors in healthy infants at 12 to 24 months of age.

**Design:**

This study included 525 healthy infants (53% girls), who participated in a randomized vitamin D intervention trial and received daily vitamin D_3_ supplementation of either 10 or 30 μg from age 2 weeks to 24 months. Biochemical parameters were measured at 12 and 24 months. Dietary phosphate intake was determined at 12 months.

**Main Outcome Measures:**

Plasma phosphate concentrations at 12 and 24 months of age.

**Results:**

Mean (SD) phosphate concentration decreased from 12 months (1.9 ± 0.15 mmol/L) to 24 months (1.6 ± 0.17 mmol/L) of age (*P* < 0.001 for repeated measurements). When adjusted by covariates, such as body size, creatinine, serum 25-hydroxyvitamin D, intact and C-terminal fibroblast growth factor 23, mean plasma phosphate was higher in boys than girls during follow-up (*P* = 0.019). Phosphate concentrations were similar in the vitamin D intervention groups (*P* > 0.472 for all). Plasma iron was associated positively with plasma phosphate at both time points (B, 0.006 and 0.005; 95% CI, 0.004-0.009 and 0.002-0.008; *P* < 0.001 at both time points, respectively). At 24 months of age, the main modifier of phosphate concentration was plasma creatinine (B, 0.007; 95% CI 0.003-0.011, *P* < 0.001).

**Conclusion:**

Plasma phosphate concentration decreased from age 12 to 24 months. In infants and toddlers, the strongest plasma phosphate modifiers were sex, iron, and creatinine, whereas vitamin D supplementation did not modify phosphate concentrations.

Although only 0.8% of the total human body weight consists of phosphate (1), it is one of the most abundant minerals in the body, and necessary for many different vital processes (2). Phosphate participates in energy metabolism, synthesis of DNA and RNA, and regulation of proteins by phosphorylation (3). Phosphate also plays a crucial role in mineral metabolism and about 85% of phosphate is present as hydroxyapatite in bone and teeth (4). Abnormalities in phosphate metabolism may cause rickets, osteomalacia, or soft-tissue mineralization (5).

Phosphate homeostasis is regulated by a complex network of factors, including PTH, 1,25-dihydroxyvitamin D (1,25(OH)2D), fibroblast growth factor 23 (FGF23), and calcitonin (6-8). The phosphatonins, secreted frizzled related protein 4, and insulin-like growth factor 1 modulate phosphate concentration by affecting the activity of 25-hydroxyvitamin D_3_-1α-hydroxylase (7,9,10). High creatinine concentration associates with high phosphate concentration in preterm infants (11), and iron administration has been found to reduce phosphate concentrations in adults (12), possibly through its effects on FGF23.

Generally, phosphate concentrations are higher in healthy infants and children than in adults (13-15). Published normative data, especially for young children, have thus far been based largely on small cohort studies, often including premature infants (16-18). Published reference values for phosphate concentration vary from 1.54 to 2.72 mmol/L for infants (aged 15 days to 1 year), 1.38 to 2.19 mmol/L for children (aged 1 to 5 years), and 1.33 to 1.92 mmol/L for older children and adolescents (aged 5-13 years) (13).

The temporal changes and factors modifying phosphate concentrations in children 12 to 24 months of age have not been previously studied. Our study examined plasma phosphate concentrations in a large cohort of healthy young Finnish children at 12 and 24 months and studied modifying factors for phosphate concentrations at these time points.

## Material and Methods

### Study participants

This study is part of the Vitamin D Intervention trial in infants (VIDI), a prospective, double-blinded and randomized intervention study, performed in Helsinki, Finland. In the VIDI trial, altogether 975 healthy infants were randomized to receive vitamin D supplementation either 10 µg (group10) or 30 µg (group30) daily from 2 weeks to 24 months of age (19). The infants were carefully monitored clinically and biochemically during the trial. The detailed VIDI protocol and inclusion and exclusion criteria as well as the main findings of the study have previously been reported (19,20). The study was conducted in accordance with the principles of The Declaration of Helsinki. Research permit was obtained from The Research Ethics Committee of the Hospital District of Helsinki and Uusimaa (107/13/03/03/2012) and all participating families gave informed consent before study onset. The trial protocol is registered in ClinicalTrials.gov (NCT01723852).

The present study includes data from a total of 525 VIDI participants for whom plasma phosphate concentrations were available at both 12 and 24 months of age. Subjects with incomplete data on phosphate concentrations (n = 152) as well as those later diagnosed with significant medical conditions (n = 8) were excluded (21). Baseline data were collected during recruitment from the participating infants’ medical records. Growth parameters and venous blood samples for analyses of biochemical parameters were obtained at follow-up visits at 12 and 24 months of age. Growth parameters were evaluated according to Finnish pediatric growth references (22). Obtained samples were stored at -80°C until completed analyses.

### Biochemical assays

Because of the participants’ young age, there was no fasting before sampling. The samples were taken between morning and early afternoon. Laboratory analyses for plasma phosphate were performed at the Central Laboratory of Helsinki University Hospital using accredited standard methodology with Cobas c311 and Cobas c501/502 based on photometric assay (Roche/Hitachi, Basel, Switzerland).

Ionized calcium, alkaline phosphatase, creatinine, and iron concentrations were determined at the Central Laboratory of Helsinki University Hospital, as previously reported (23). Serum 25-hydroxyvitamin D (25OHD) and PTH were analyzed using IDS-iSYS fully automated immunoassay system (Immunodiagnostic Systems, Ltd., Bolton, UK) with chemiluminescence detection at the Pediatric Research Centre, University of Helsinki (19).

Participation in the vitamin D External Quality Assessment Scheme (Charing Cross Hospital, London, UK) guaranteed the quality and accuracy of the vitamin D assay. Both intact and C-terminal FGF23 concentrations were determined from plasma samples by immunosorbent assays (Kainos Laboratories, Tokyo, Japan, for intact FGF23 and Biomedica Medizinprodukte GmbH & Co KG, Vienna, Austria, for C-terminal FGF23) in the laboratory of the Pediatric Research Center, University of Helsinki, as reported previously (34).

### Nutritional data

Nutritional data on food consumption were obtained from 3-day food diaries, including 2 weekdays and 1 weekend day, as previously described (24). Dietary intake data at 12 months of age were given by the parents or daycare personnel and they were analyzed using AivoDiet software (version 2.0.2.3, Aivo Oy, Turku, Finland). Relative phosphate intake was calculated as daily total phosphate intake relative to daily total energy intake.

### Statistical analysis

Results are presented as mean and standard deviation or as median and interquartile range, as appropriate. Comparisons between sexes were performed using Student *t* test for parametric and Mann-Whitney *U* test for nonparametric variables. Chi-squared test was used for categorical variables. The dependence between variables was examined using Pearson’s correlation.

Normal distribution of the variables was primarily examined by visual assessment of histograms, assessment of Skewness and Kurtosis, and secondarily by Kolmogorov-Smirnov test. Logarithmic transformation was performed for the variables that were not normally distributed. Season was determined, by month of the study visit and sampling, as winter (December, January, and February), spring (March, April, and May), summer (June, July, and August), and fall (September, October, and November). Season was used as a dichotomous variable in analyses (1 = winter, 2 = others) because concentrations of phosphate in winter were higher than at other times of the year, in post hoc analyses.

The effects of various factors on phosphate concentration were studied using a linear regression model (forward method, including variables with a *P* value < 0.05). The covariates were selected based on correlation and previous studies related on phosphate (7,8,11,12,25).

The differences in phosphate levels between the sexes and intervention groups and changes over time were studied using a mixed model with a diagonal covariance structure with heterogeneous variance. Based on Akaike’s information criterion, this default covariance structure for repeated measures performed better than other options.

The mixed models included the covariates with a *P* value < 0.05 and took into account the size of the children (weight, length). Estimation method was restricted maximum likelihood.

Statistical analyses were performed with IBM SPSS Statistics 25 (IBM, Armonk, NY, USA). *P* values < 0.05 were considered statistically significant.

## Results

### Characteristics of participating children

Characteristics of the participating infants at 12 and 24 months are presented in Table 1.

**Table 1.** General, biochemical, and nutritional characteristics at 12 and 24 months of age

	Boys	Girls	*P* value (*t* test)
Participants	245	280	
Group_10_ vs group_30_	120 vs 125	137 vs 143	
Pregnancy (d)	281 ± 7.6	282 ± 7.0	0.112
**At 12 months of age**			
Length (cm)	76.0 ± 5.39	74.6 ± 2.27	**<0.001**
Weight (kg)	10.2 ± 1.10	9.4 ± 0.99	**<0.001**
Season (n)^*a*^			0.491^*b*^
Winter	19.2% (n = 47)	17.9% (n = 50)	
Spring	41.2% (n = 101)	45.0% (n = 126)	
Summer	20.8% (n = 51)	22.9% (n = 64)	
Autumn	18.8% (n = 46)	14.3% (n = 40)	
**Biochemical variables**			
Phosphate (mmol/L)	1.9 ± 0.15	1.9 ± 0.16	0.416
25OHD (nmol/L)	100 ± 29.5	101 ± 29.3	0.697
Ionized calcium (mmol/L)^*c*^	1.33 ± 0.031	1.34 ± 0.033	**<0.001**
Iron (µmol/L)	10.9 ± 4.78	11.1 ± 5.03	0.646
Creatinine (µmol/L)	25.6 ± 6.15	26.0 ± 6.71	0.548
PTH (pg/mL)	23.1 (15.28, 31.20)	24.3 (15.80, 34.50)	0.321^*d*^
Alkaline phosphatase (U/L)	287 (239.3, 340.5)	270 (219.0, 340.5)	0.113^*d*^
Intact FGF23 (pg/mL)	40.6 (34.41, 48.95)	44.9 (37.32, 51.53)	**<0.001** ^*d*^
C-terminal FGF23 (pmol/L)	2.8 (2.20, 3.71)	2.9 (2.24, 3.62)	0.722^*d*^
**Dietary intake** ^*d*^			
Phosphate (mg/d)	734 ± 281.9	690 ± 263.7	0.081
Calcium (mg/d)	624 ± 323.3	597 ± 308.2	0.352
Iron (mg/d)	6.4 ± 2.22	5.9 ± 1.94	**0.017**
Vitamin D (mcg/d)	6.0 ± 3.42	6.0 ± 3.41	0.994
Energy (kcal/d)	808 ± 218.3	768 ± 197.4	**0.040**
**At 24 months of age**			
Length (cm)	88.5 ± 2.94	87.1 ± 2.84	**<0.001**
Weight (kg)	12.8 ± 1.30	12.2 ± 1.26	**<0.001**
Season (n)^*a*^			0.595^*b*^
Winter	22.4% (n = 55)	21.1% (n = 59)	
Spring	39.6% (n = 97)	44.3% (n = 124)	
Summer	21.2% (n = 52)	21.4% (n = 60)	
Autumn	16.7% (n = 41)	13.2% (n = 37)	
**Biochemical variables**			
Phosphate (mmol/L)	1.6 ± 0.16	1.6 ± 0.17	0.150
25OHD (nmol/L)	102 ± 27.6	104 ± 29.1	0.501
Ionized calcium (mmol/L)^*c*^	1.30 ± 0.032	1.31 ± 0.032	**0.002**
Iron (µmol/L)	12.5 ± 5.13	13.3 ± 5.15	0.076
Creatinine (µmol/L)	23.8 ± 5.05	23.5 ± 4.31	0.395
PTH (pg/mL)	16.2 (10.70, 21.80)	16.9 (11.45, 23.75)	0.099^*d*^
Alkaline phosphatase (U/L)	246 (208.3, 290.0)	235 (197.0, 281.0)	0.116^*d*^
Intact FGF23 (pg/mL)	38.8 (33.02, 45.81)	43.6(36.11, 50.42)	**<0.001** ^*d*^
C-terminal FGF23 (pmol/L)	1.9 (1.53, 2.71)	1.9 (1.48, 2.49)	0.499^*d*^

Values are given as means ± SD, or as median and interquartile range (IQR), or as % (n). *P* values are determined between the sexes at 12 and 24 months of age. Reference range: PTH 15-70 pg/mL, ionized calcium 1.17-1.35 mmol/L, phosphate 1.3-2.2 mmol/L, iron 7-28 μmol/L, ferritin: 6-60 μg/L, hemoglobin 112-142 g/L.

Abbreviations: 25OHD, serum 25-hydroxyvitamin D; FGF23, fibroblast growth factor 23.

^*a*^Winter = December, January, February; spring = March, April, May; summer = June, July, August; autumn = September, October, November.

^*b*^Pearson χ ^2^ test.

^*c*^n = 525, total number of n is missing 5 subjects or more: ionized calcium at 12 mo, n = 520; dietary intake variables, n = 460; ionized calcium at 24 mo, n = 490; alkaline phosphatase at 24 mo, n = 518, intact FGF23 at 12 mo, n = 515; C-terminal FGF23 at 12 mo, n = 509.

^*d*^Mann-Whitney *U* test.

The study included 525 infants (53% girls). Boys were longer and heavier than girls at both 12 and 24 months of age (*t* test *P* < 0.001 at both time points) (Table 1).

Mean unadjusted calcium concentrations in girls were slightly higher than in boys at age 12 months and 24 months (*t* test *P* < 0.001 and *P* = 0.002, respectively) (Table 1). PTH concentrations did not differ between sexes at 12 and 24 months (Mann-Whitney *U* test *P* > 0.099 for all) (Table 1). As previously reported (34), intact FGF23 concentrations were higher in girls than in boys at 12 and 24 months of age (Mann-Whitney *U* test *P* < 0.001 for all), whereas C-terminal FGF23 concentration did not differ between the sexes (Mann-Whitney *U* test *P* > 0.499 for all) (Table 1). Serum 25OHD was between 50 and 125 nmol/L in 80.9% of the participants at 12 months and in 78.1% at 24 months of age. Fewer than 1.1% of the participants had serum 25OHD < 50 nmol/L at both time points. In the remaining children, 25OHD was > 125 nmol/L.

One-third of the infants were partially breastfed at 12 months of age (24). Boys received more energy (kcal/d) and iron than girls (*t* test *P* < 0.040 for all) (Table 1).

### Circulating phosphate concentration

**Figure 1.**
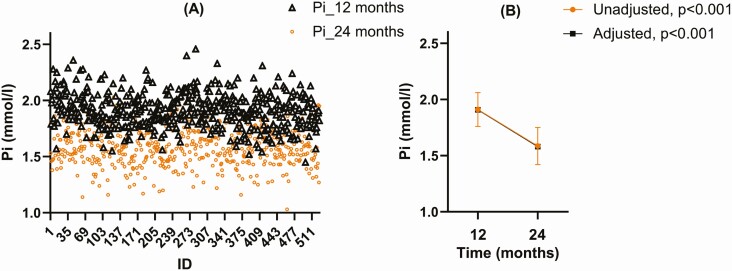
(A) Phosphate concentrations in children 12 and 24 months of age according to study identification number (ID). (B) Mean of phosphate concentrations in children at 12 and 24 months of age (unadjusted: mean ± SD; adjusted: estimates mean ± SE). Unadjusted mixed model with repeated measurements without covariates (*P*_interaction_ < 0.001) and adjusted mixed model with repeated measurements with covariates (*P*_interaction_ < 0.001): season, iron, length, weight, creatinine, ionized calcium, 25OHD, C-terminal FGF23, and intact FGF23. *P*_interaction_ indicates repeated measurements of covariance.

Mean unadjusted phosphate concentrations were at 12 months 1.9 ± 0.15 mmol/L in boys and 1.9 ± 0.16 mmol/L in girls, and at 24 months 1.6 ± 0.15 mmol/L in boys and 1.6 ± 0.17 mmol/L in girls. The phosphate concentrations for all study participants at 12 and 24 months of age are presented in Fig. 1.

Unadjusted phosphate concentrations were largely (90.1% and 99.8% at 12 and 24 months, respectively) within the previously reported age-related reference range (1.25-2.10 mmol/L) (26). However, at 12 months, phosphate concentrations were above the reference range in 9.9% of the children (n = 52). At 24 months, 0.2% of the values (n = 1) were above and 2.3% (n = 12) below the reference values. Unadjusted phosphate concentrations according to sex and the vitamin D intervention group are presented in Table 2.

**Table 2.** Phosphate concentrations according to sex and intervention group at 12 and 24 months

				Percentiles				
			Range	5	25	50	75	95
Sex	Boys	12 mo	1.55-2.36	1.69	1.82	**1.90**	2.00	2.19
	(n = 245)	24 mo	1.14-2.03	1.31	1.47	**1.57**	1.69	1.83
	Girls	12 mo	1.45-2.46	1.65	1.80	**1.91**	2.01	2.15
	(n = 280)	24 mo	1.03-2.13	1.32	1.49	**1.59**	1.71	1.88
Intervention group	Group10	12 mo	1.45-2.28	1.66	1.82	**1.91**	2.01	2.17
	(n = 257)	24 mo	1.19-2.03	1.34	1.50	**1.58**	1.69	1.86
	Group30	12 mo	1.52-2.46	1.65	1.81	**1.91**	2.01	2.17
	(n = 268)	24 mo	1.03-2.13	1.27	1.46	**1.57**	1.70	1.86

Group10 or Group30 infants received daily vitamin D supplementation 10 µg (group10) or 30 µg (group30) from 2 wk to 24 mo of age. Median values are marked in bold.

**Figure 2.**
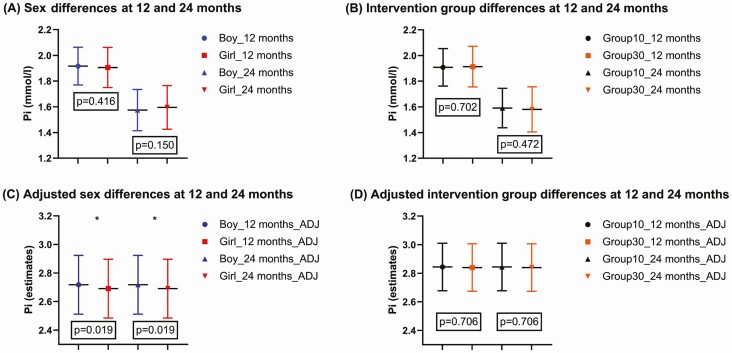
Differences in mean phosphate concentrations (mmol/L) between sexes and intervention groups (mean ± SD, estimates mean ± SE [adj]). (A) Phosphate concentrations (mmol/L) at 12 months of age between girls and boys (*t* test *P* = 0.416) and at 24 months (*t* test *P* = 0.150). (B) Phosphate concentrations (mmol/L) at 12 months of age between intervention groups (*t* test *P* = 0.702) and at 24 months (*t* test *P* = 0.472). (C) Phosphate concentrations (estimates) at 12 months and 24 months of age between girls and boys (mixed model, Bonferroni *P* = 0.019). (D) Phosphate concentrations (estimates) at 12 and 24 months of age between the vitamin D intervention groups (mixed model, Bonferroni *P* = 0.706). Adjusted values were obtained by the analysis of mixed model with time-dependent covariates. The covariates were season (1 = winter, 2 = others), iron (µmol/L), length (cm), weight (kg), creatinine (µmol/L), ionized calcium (mmol/L), 25OHD (nmol/L), C-terminal FGF23 (pmol/L), and intact FGF23 (pg/mL).

In unadjusted models, phosphate concentrations did not differ between the sexes (*t* test *P* = 0.416 and *P* = 0.150 at 12 and 24 months, respectively) (Table 1). However, mixed model analyses for repeated measurements showed a difference in phosphate concentrations between the sexes at 24 months of age with boys having higher phosphate concentrations than girls (mean difference 0.027, mixed model, Bonferroni *P* = 0.019) (Fig. 2). The mixed models were adjusted by season, iron, length, weight, creatinine, ionized calcium, 25OHD, C-terminal FGF23, and intact FGF23.

No differences in phosphate concentrations were observed between the vitamin D intervention groups, when analyzed in both sexes together (*t* test *P* = 0.702 and 0.472 at 12 and 24 months, respectively) (Fig. 2) or separately for boys and girls (post hoc test, Bonferroni *P* = 0.558 and 0.574 at 12 months of age, and *P* = 0.075 and 0.786 at 24 months of age, for group10 and group30, respectively) (Table 3). Similarly, in adjusted analysis the intervention group did not affect phosphate concentrations (mixed model, Bonferroni *P* = 0.706) (Fig. 2).

**Table 3.** Basic statistical parameters of phosphate and phosphate intake categorized by sex and intervention group

		Group10			Group30		
		Boys	Girls		Boys	Girls	
		120	137	*P* ^*a*^	125	143	*P* ^*a*^
At 12 months of age							
Phosphate (mmol/L)	Mean ± SE	1.91 ± 0.01	1.90 ± 0.01	0.558	1.92 ± 0.01	1.91 ± 0.01	0.574
	Range (min-max)	1.55-2.28	1.45-2.21	-	1.56-2.36	1.52-2.46	-
Phosphate intake (mg/d)	Mean ± SE	769 ± 26.0	681 ± 25.5	**0.015**	703 ± 26.5	699 ± 23.4	0.915
	Range (min-max)	73-1466	176-1371	-	209-1650	94-1277	-
At 24 months of age							
Phosphate (mmol/L)	Mean ± SE	1.57 ± 0.01	1.61 ± 0.01	0.075	1.58 ± 0.01	1.58 ± 0.02	0.786
	Range (min-max)	1.19-2.03	1.27-2.02	-	1.14-1-96	1.03-2.13	-

Group10 or group30 infants received daily vitamin D supplementation 10 µg (group10) or 30 µg (group30) from 2 wk to 24 mo of age.

^*a*^ *P* value (Bonferroni).

### Phosphate intake and temporal change

Dietary intake of phosphate at 12 months was similar in girls and boys (*t* test *P* = 0.081) (Table 1). When studied by intervention group, phosphate intake was higher in boys than in girls in group10 (post hoc test, Bonferroni *P* = 0.015) (Table 3).

Because unadjusted phosphate concentrations did not differ between the sexes or intervention groups at 12 or 24 months, analyses of temporal change were performed on the whole study population, and not separately by sex or intervention group. Mean phosphate concentrations decreased from 1.9 ± 0.15 mmol/L at 12 months to 1.6 ± 0.17 mmol/L at 24 months of age without covariates (*P*_interaction_ < 0.001) and by 0.33 mmol/L units with covariates (*P*_interaction_ < 0.001) (Fig. 1). The covariates of time and weight were statistically significant (Wald *Z P* < 0.001 for both), but length was not significant on the mixed model of time-repeated measures. Other covariates in the model were season, creatinine, ionized calcium, iron, 25OHD, C-terminal, and intact FGF23.

### Factors modifying phosphate concentration

Iron-modified phosphate concentrations positively at both studied time points (B, 0.006 and 0.005; 95% CI, 0.004-0.009 and 0.002-0.008, *P* < 0.001 at both 12 and 24 months, respectively) (Table 4). At 12 months of age calcium intake from food was positively associated with phosphate concentrations (B, 0.047; 95% CI, 0.006-0.089, *P* = 0.027) and season was found to modify phosphate, with higher concentrations observed in winter (Bonferroni *P* = 1.000, 0.004, and 0.154 for winter vs spring, winter vs summer, and winter vs autumn, respectively) than other seasons (B, -0.040; 95% CI -0.075 to -0.006; *P* = 0.022) (Table 4). PTH did not modify phosphate concentrations at either 12 or 24 months of age. Relative phosphate intake from food at 12 months of age did not correlate with circulating phosphate concentration (Pearson’s correlation, *r* = 0.077, *P* = 0.100). At 24 months of age, but not at 12 months, C-terminal FGF23 and creatinine positively associated with phosphate concentration (B, 0.036 and 0.010; 95% CI, 0.007-0.065 and 0.006-0.013; *P* = 0.016; *P* < 0.001, respectively) (Table 4).

**Table 4.** Modifying factors of phosphate concentrations at 12 and 24 months of age

Variable	β	B	*t*	*P* value
**Phosphate concentration at 12 mo of age (mmol/L)**				
Iron, µmol/L	0.210	0.006	4.527	<0.001
Season (1 = winter, 2 = others)	-0.106	-0.040	-2.301	0.022
Calcium in food (mg/kcal)	0.103	0.047	2.225	0.027
**Phosphate concentration at 24 mo of age (mmol/L)**				
Creatinine (µmol/L)	0.268	0.010	6.170	<0.001
Iron, µmol/L	0.155	0.005	3.524	<0.001
C-terminal FGF23, pmol/L	0.107	0.036	2.419	0.016

The following variables were included in the regression model at 12 months of age: sex, season, 25OHD, ionized calcium, creatinine, dietary intake variables (phosphate, calcium, vitamin D), iron, alkaline phosphatase,^*a*^ PTH,^*a*^ length, weight, intact FGF23,^*a*^ and C-terminal FGF23.^*a*^ Model at 12 months of age: *R*^2^ 0.068, adjusted *R*^2^ 0.062, significant F change 0.027, Durbin-Watson 2.069. The following variables were included in the regression model at 24 months of age: sex, season, 25OHD, ionized calcium, creatinine, iron, alkaline phosphatase, PTH, length, weight, intact FGF23,^*a*^ and C-terminal FGF23.^*a*^ Model at 24 months of age: *R*^2^ 0.101, adjusted *R*^2^ 0.096, significant F change 0.016, Durbin-Watson 1.924. Analysis performed using linear regression model with forward method. The table includes all variables with statistical significance below 0.05 (*P* < 0.05).

Abbreviations: 25OHD, serum 25-hydroxyvitamin D; β, standardized regression coefficient; B, regression coefficient; FGF23, fibroblast growth factor 23; *t*, t-statistic.

^*a*^ = after logarithmic transformation.

When stratified by sex, iron-modified phosphate concentrations positively at 12 months of age in both boys and girls (B, 0.004 and 0.007; 95% CI, < 0.001-0.008 and 0.003-0.011, *P* = 0.033 and *P* < 0.001, respectively) (Table 5). In boys, phosphate concentrations were also modified positively by intact FGF23 (B, 0.073; 95% CI, 0.012-0.134; *P* = 0.020), 25OHD (B, -0.001; 95% CI, -0.001 to < 0.001; *P* = 0.024) and calcium intake from food (B, 0.062; 95% CI, 0.001-0.122; *P* = 0.045). In girls, season was associated with phosphate, concentrations being highest during winter (B, -0.064; 95% CI, -0.112 to -0.016; *P* = 0.009) (Table 5).

**Table 5.** Modifying factors of phosphate concentration stratified by sex at 12 months of age

Variable	β	B	*t*	*P* value
**Boys**				
Iron, µmol/L	0.143	0.004	2.142	0.033
Intact FGF23 (pg/mL)	0.157	0.073	2.352	0.020
25OHD (nmol/L)	-0.149	-0.001	-2.272	0.024
Calcium in food (mg/kcal)	0.134	0.062	2.018	0.045
**Girls**				
Iron, µmol/L	0.244	0.007	3.790	<0.001
Season (1 = winter, 2 = others)	-0.170	-0.064	-2.644	0.009

The following variables were included in the regression model: season, PTH,^*a*^ 25OHD, ionized calcium, alkaline phosphatase,^*a*^ iron, creatinine, dietary intake variables (phosphate, calcium, vitamin D), length, weight, intact FGF23,^*a*^ and C-terminal FGF23.^*a*^ Model (boys): *R*^2^ 0.091, adjusted *R*^2^ 0.074, significant F change 0.045, Durbin-Watson 2.136. Model (girls): *R*^2^ 0.091, adjusted *R*^2^ 0.082, significant F change 0.009, Durbin-Watson 2.030. Analysis performed using linear regression model with forward method. The table includes all variables with statistical significance below 0.05 (*P* < 0.05).

Abbreviations: 25OHD, serum 25-hydroxyvitamin D; β, standardized regression coefficient; B, regression coefficient; FGF23, fibroblast growth factor 23; *t*, t-statistic.

^*a*^After logarithmic transformation.

At 24 months of age, plasma creatinine was a key modifying factor in both sexes (B, 0.007 and 0.013; 95% CI, 0.003-0.011 and 0.008-0.017; *P* < 0.001 for both) (Table 6). Creatinine correlated negatively with ionized calcium (Pearson’s correlation, *r* = -0.092, *P* = 0.042) and dietary intake variables, such as phosphate, iron, calcium, and vitamin D (Pearson’s correlation, *r* < -0.090, *P* > 0.055 for all) and positively with PTH (Pearson’s correlation, *r* = 0.098, *P* = 0.024, after logarithmic transformation). At 24 months, iron and C-terminal FGF23 were modifying factors in boys (B, 0.006 and 0.046; 95% CI, 0.002-0.010 and 0.004-0.088; *P* = 0.004 and *P* = 0.031, respectively) but not in girls (Table 6). Length or PTH did not modify phosphate concentrations at either 12 or 24 months of age (Tables 4, 5, and 6).

**Table 6.** Modifying factors of phosphate concentration stratified by sex at 24 months of age

Variable	β	B	t	*P* value
**Boys**				
Creatinine, µmol/L	0.226	0.007	3.540	<0.001
Iron, µmol/L	0.190	0.006	2.945	0.004
C-terminal FGF23, pmol/L	0.140	0.046	2.175	0.031
**Girls**				
Creatinine, µmol/L	0.317	0.013	5.326	<0.001

The following variables were included in the regression model: season, 25OHD, ionized calcium, iron, creatinine, PTH,^*a*^ alkaline phosphatase,^*a*^ length, weight, intact FGF23,^*a*^ and C-terminal FGF23.^*a*^ Model (boys): *R*^2^ 0.098, adjusted *R*^2^ 0.086, significant F change 0.031, Durbin-Watson 1.811. Model (girls): *R*^2^ 0.100, adjusted *R*^2^ 0.097, significant F change < 0.001, Durbin-Watson 2.023. Analysis performed using linear regression model with forward method. The table includes all variables with statistical significance below 0.05 (*P* < 0.05).

Abbreviations: 25OHD, serum 25-hydroxyvitamin D; β, standardized regression coefficient; B, regression coefficient; FGF23, fibroblast growth factor 23; *t*, t-statistic.

^¥^ = after logarithmic transformation.

## Discussion

To the best of our knowledge, this is the first study to investigate phosphate concentrations and modifying factors in healthy infants aged 12 to 24 months. Our comparatively large study population (n = 525) and the longitudinal study setting allowed us to evaluate factors influencing changes in phosphate homeostasis in this age group. The current study shows that children have significantly higher phosphate concentrations at age 12 months than at 24 months. No statistically significant difference in phosphate concentrations was observed between the vitamin D intervention groups (10 µg vs 30 µg), and the primary modifying factors for plasma phosphate concentrations were plasma iron and creatinine concentrations that both associate positively with phosphate concentration.

We observed a significant decrease in phosphate concentration from age 12 months to 24 months. We did not find previous similar observations in the literature. Adeli et al. studied phosphate concentrations in children and adolescents aged 0 to 19 years, and 368 children aged 1 to 5 years participated in this cross-sectional study (13). However, their cross-sectional study did not specifically compare phosphate concentrations between different age groups in early childhood. Our results indicate that major physiological changes in phosphate homeostasis take place also after the first year of life. Our study did not evaluate potential molecular mechanisms behind these observations. One possible explanation of higher phosphate concentrations at age 12 months can be age-dependent changes in expression levels of sodium-phosphate cotransporters. The expression of genes and proteins responsible for phosphate absorption decreases with age, and this could also contribute to lower phosphate levels in 24-month-old children compared with 12-month-old children (27).

Unadjusted phosphate concentrations, or temporal change from 12 to 24 months, did not differ by sex. In the adjusted model for repeated measurements, boys had higher phosphate concentrations than girls at 12 and 24 months, and the difference would be explained by the selection of covariates. No sex differences in phosphate concentrations were observed in children aged 1 to 5 years in the Caliper study, which also supports our results (13). In a large cohort of adults including overweight, hypertensive, and diabetic patients (n = 92 756), unadjusted phosphate concentrations were higher in women than in men (28). Age (young children vs adults) and cohort selection (healthy children vs adults with a chronic illness) could potentially explain the differences between these findings. Possible age- and sex-dependent differences in phosphate concentrations and modifying factors during childhood warrant further investigation.

We observed no differences in phosphate concentrations between vitamin D intervention groups. Vitamin D plays a crucial role in calcium and phosphate metabolism and is essential for bone health in infants, children, and adolescents (29). Low phosphate concentration is a common finding in children with rickets (5,30,31) or vitamin D deficiency (serum 25OHD < 50 nmol/L) (30), most likely because of increased phosphate excretion in response to secondary hyperparathyroidism. In our cohort, 25OHD levels were largely normal and this is a likely reason why no association between vitamin D or PTH and phosphate concentrations was detected.

In our study, 99.8% of phosphate concentrations at 24 months were within the previously described reference range (1.25-2.10 mmol/L aged 1-3 years), whereas at 12 months almost 10% of the values were above these references (26). In previous studies, reference values have not been specified separately for 12 and 24 month olds (13,26). Our results, showing that phosphate concentrations decrease from 12 to 24 months, indicate that age-specific reference ranges should be used in young children in order to correctly early identify infants with subnormal or supranormal phosphate values. Our data can potentially be used to update reference values in this age-group.

In our study, iron was the main modifying factor of phosphate concentrations at 12 months and modified phosphate concentrations also at 24 months. Previous research on the association of iron and phosphate concentrations is limited. Our findings indicate that iron is positively associated with phosphate concentrations at 12 and 24 months of age. In contrast, previous studies have shown iron administration to result in a transient decrease in phosphate (12,32). The effect depends on the type of iron: ferric carboxymaltose induces hypophosphatemia, whereas ferric dextran does not have a similar effect. However, in an experiment in rats, when a high dose of iron was administered, no difference in phosphate concentrations was observed (33). We have previously reported that iron is an important modifying factor of FGF23 in healthy infants, with iron being positively associated with intact FGF23 and inversely associated with C-terminal FGF23 (23,34). In the present study, intact and C-terminal FGF23 showed variable association with phosphate, depending on the time point and sex. As FGF23 is a phosphate modifying factor, it seems that iron regulates phosphate metabolism through FGF23. Our study confirms the existence of the link between iron and phosphate metabolism already in early childhood. Further research on specific mechanisms of this regulation is needed.

Creatinine was the second major modifying factor of phosphate at 24 months of age. In our study, creatinine concentration was positively associated with phosphate concentration. The association between phosphate and creatinine clearance is well known in patients with renal failure, creatinine being a marker of kidney function (35). Anthropometric measurements, including arm circumference, have been linked to creatinine in fully active children aged 2 to 6 years, so creatinine may associate with muscle mass growth also in young children (36). However, our study found no statistically significant correlation between creatinine and anthropometric variables.

Other significant modifying factors were 25OHD and season. As previously published, seasonal fluctuations have been observed in phosphate concentrations (37); this was also seen in our models. In our study, children’s phosphate levels were slightly higher in winter than in other seasons, but the differences between seasons were minimal. As previously speculated, the seasonal variation of phosphate comes through vitamin D metabolism, particularly via 1,25(OH)2D (38). In our study, seasonal variation in PTH concentrations was also observed at 24 months of age. Possible explanations for the phenomenon in this age group are the use of protecting clothing against UV-B radiation and staying indoors in the summertime, which may reduce the effect of seasonal variation on vitamin D status in young children (39). There is also evidence of seasonal variation in longitudinal growth in infants (40), which may explain slightly higher phosphate levels in winter when bone growth and mineralization are reduced. PTH reduces phosphate reabsorption in the proximal renal tubules and is therefore known to be involved in phosphate regulation. Surprisingly, PTH was not a significant modifying factor of phosphate concentration in the models used in this study.

Calcium and phosphate play key roles in skeletal development (41). In our findings, relative dietary calcium intake was positively associated with phosphate concentration in children. However, Jafari Giv et al. did not find a relationship between dietary calcium and phosphate intake and serum calcium and phosphate levels (42). In our cohort, relative dietary phosphate intake correlated weakly with phosphate concentrations. Based on this and previously published results (42), dietary phosphate correlates poorly with circulating phosphate levels. The children’s weight also contributed to the phosphate variance in the mixed model, but this finding does not suggest that a relatively greater weight gain would result in a relatively larger decrease in phosphate levels in early childhood.

The strengths of our study are a large research cohort (n = 525) of healthy infants and follow-up at 2 different time points (12 and 24 months of age), allowing longitudinal analyses. The vitamin D intervention study gave us an opportunity to evaluate also how vitamin D affected the results. Our study was limited by lack of more complete data on phosphate modifying factors because small total volume of blood samples prevented us from studying additional parameters, such as 1,25(OH)2D or other phosphatonins that might have had an effect of phosphate concentration. Further, measurement of urinary excretion of phosphate, and tubular maximum reabsorption of phosphate/glomerular filtration rate, and nutritional data at 24 months of age could have provided additional information on phosphate metabolism and factors modifying phosphate concentration in these young children.

## Conclusion

Our study presents normative phosphate concentrations in healthy children aged 12 to 24 months. The observed decrease in phosphate concentrations from 12 to 24 months is a novel finding. Iron at both time points, and creatinine at 24 months, were the key modifiers associated positively with phosphate concentrations. Vitamin D supplementation did not modify phosphate concentrations, but sex and intact and C-terminal FGF23 may affect phosphate concentrations. The reported normative data should prove useful for early detection of children with hypo- or hyperphosphatemia. Further research on phosphate metabolism in early childhood is needed.

## Data Availability

Restrictions apply to the availability of some or all data generated or analyzed during this study to preserve patient confidentiality or because they were used under license. The corresponding author will on request detail the restrictions and any conditions under which access to some data may be provided.

## Abbreviations

1,25(OH)2D: 1,25-dihydroxyvitamin D
25OHD: serum 25-hydroxyvitamin D
FGF23: fibroblast growth factor 23;
VIDI: Vitamin D intervention in infants.

## Additional Information

***Disclosures:*** O.M. declares consultancy to Kyowa Kirin, Alexion, Merck, and Sandoz. The other authors declare no conflicts of interest.
